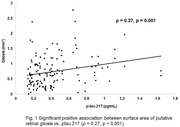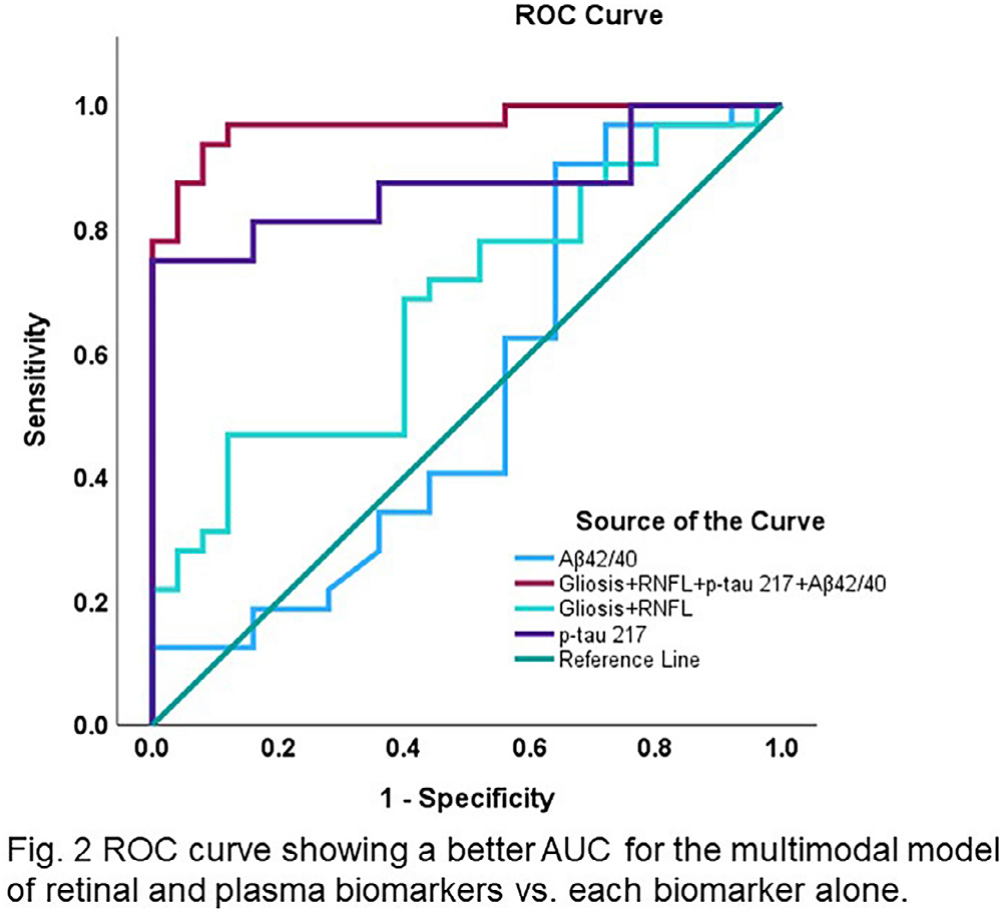# Association and multimodal model of retinal and blood‐based biomarkers for detection of preclinical Alzheimer’s disease

**DOI:** 10.1002/alz.084715

**Published:** 2025-01-09

**Authors:** Swetha Ravichandran, Peter J. Snyder, Jessica Alber, Andreas Jeromin, Lauren E Chaby, Edmund Arthur

**Affiliations:** ^1^ University of Alabama at Birmingham, Birmingham, AL USA; ^2^ Alpert Medical School of Brown University, Providence, RI USA; ^3^ University of Rhode Island, Kingston, RI USA; ^4^ Butler Hospital Memory and Aging Program, Providence, RI USA; ^5^ George & Anne Ryan Institute for Neuroscience, Kingston, RI USA; ^6^ ALZpath, Inc., Carlsbad, CA USA

## Abstract

**Background:**

The potential of plasma Aβ42/Aβ40 ratio, NfL, p‐tau 181, and p‐tau 217 has been extensively discussed in the literature. Our previous study explored the association between retinal biomarkers and preclinical AD. The goal of this study was to evaluate the association and a multimodal model of retinal and plasma biomarkers for detection of preclinical AD.

**Methods:**

We included 81 cognitively unimpaired (CU) participants (139 eyes; mean age: 67 years; range: 56‐80) from the Atlas of Retinal Imaging in Alzheimer's Study (ARIAS). Blood samples were assessed for concentrations of Aβ42/Aβ40 ratio, NfL, p‐tau 181, and p‐tau 217 (ALZpath, Inc.) using SIMOA technology. Spectralis II instrument was used to acquire macular centered SD‐OCT images for evaluation of putative retinal gliosis surface area and macular retinal nerve fiber layer (RNFL) thickness. A subgroup cohort of 57 eyes (32 preclinical, 25 controls; mean age: 69 vs 66, p = 0.06) were included for assessment of a multimodal model to distinguish between preclinical AD (PET Aβ+ve) and controls (PET Aβ‐ve). For all participants, partial correlations (accounting for age) were assessed between retinal and blood‐based biomarkers. For the subgroup cohort, an ROC analysis was performed to compare a multimodal model of the retinal and plasma biomarkers vs. each biomarker alone to distinguish between the two groups.

**Result:**

Significant positive partial correlation was found between putative retinal gliosis and p‐tau 217 (ρ = 0.27, p = 0.001) but not for the other plasma biomarkers. The multimodal ROC model based on retinal (gliosis area, inner inferior RNFL thickness, inner superior RNFL thickness, and inner nasal RNFL thickness) and plasma biomarkers (p‐tau 217 and Aβ42/Aβ40 ratio) had an excellent AUC of 0.97 (95% CI = 0.93 – 1.01; p < 0.001) compared to unimodal models of gliosis and RNFL thickness; AUC = 0.68 (95% CI = 0.54 – 0.82, p = 0.01), ptau 217; AUC = 0.87 (95% CI = 0.78 ‐ 0.97, p < 0.001), and Aβ42/Aβ40 ratio; AUC = 0.54 (95% CI = 0.38 – 0.70, p = 0.67).

**Conclusion:**

Our analysis shows the potential of integrating retinal and blood‐based biomarkers for improved detection and screening of preclinical AD.